# Mitochondrial sub-cellular localization of cAMP-specific phosphodiesterase 8A in ovarian follicular cells

**DOI:** 10.1038/s41598-019-48886-8

**Published:** 2019-08-28

**Authors:** Amel Lounas, Nathalie Vernoux, Marc Germain, Marie-Eve Tremblay, François J. Richard

**Affiliations:** 1 Centre de recherche en reproduction, développement et santé intergénérationnelle (CRDSI), Département des sciences animales, Faculté des Sciences de l’agriculture et de l’alimentation, Université Laval, Québec, Québec G1V 0A6 Canada; 2 Centre de recherche du CHU de Québec-Université Laval, Axe Neurosciences, Département de médecine moléculaire, Université Laval, Québec, Québec G1V 4G2 Canada; 3 Département de biologie médicale, Université du Québec à Trois-Rivières, Québec, G8Z 4M3 Canada

**Keywords:** Infertility, Checkpoint signalling

## Abstract

Cyclic adenosine monophosphate (cAMP) is a ubiquitous secondary messenger that plays a central role in endocrine tissue function, particularly in the synthesis of steroid hormones. The intracellular concentration of cAMP is regulated through its synthesis by cyclases and its degradation by cyclic nucleotide phosphodiesterases (PDEs). Although the expression and activity of PDEs impact the specificity and the amplitude of the cAMP response, it is becoming increasingly clear that the sub-cellular localization of PDE emphasizes the spatial regulation of the cell signalling processes that are essential for normal cellular function. We first examined the expression of PDE8A in porcine ovarian cells. PDE8A is expressed in granulosa cells, cumulus cells and oocytes. Second, we assessed the mitochondrial sub-cellular localization of PDE8A. Using western blotting with isolated mitochondrial fractions from granulosa cells and cumulus-oocyte complexes revealed immuno-reactive bands. PDE assay of isolated mitochondrial fractions from granulosa cells measured specific PDE8 cAMP-PDE activity as PF-04957325-sensitive. The immune-reactive PDE8A signal and MitoTracker labelling co-localized supporting mitochondrial sub-cellular localization of PDE8A, which was confirmed using immuno-electron microscopy. Finally, the effect of PDE8 on progesterone production was assessed during the *in-vitro* maturation of cumulus-oocyte complexes. Using PF-04957325, we observed a significant increase (P < 0.05) in progesterone secretion with follicle-stimulating hormone (FSH). Active mitochondria stained with MitoTracker orange CMTMRos were also increased by the specific PDE8 inhibitor supporting its functional regulation. In conclusion, we propose the occurrence of mitochondrial sub-cellular localization of PDE8A in porcine granulosa cells and cumulus cells. This suggests that there is potential for new strategies for ovarian stimulation and artificial reproductive technologies, as well as the possibility for using new media to improve the quality of oocytes.

## Introduction

Cyclic adenosine monophosphate (cAMP) is a ubiquitous secondary messenger that is synthesized in response to the stimulation of G-protein-coupled receptors that mediate a wide variety of important cellular functions. The functions that are mediated by cAMP include those that occur in ovarian follicular cells following the stimulation of the follicle-stimulating hormone (FSH) receptor^1^ and the luteinizing hormone (LH) receptor^2^. These functions include steroidogenesis, folliculogenesis, cell division, ovulation, oocyte maturation, cumulus cell expansion and luteinisation^3^.

The intracellular concentration of cAMP is regulated through its synthesis by adenylyl cyclases and its degradation by phosphodiesterases (PDEs). PDEs hydrolyze 3′-5′ cyclic adenosine monophosphate to produce an inactive 5′ adenosine monophosphate. PDEs belong to a superfamily of metalophosphohydrolases that are encoded by 21 genes and grouped into 11 families, based on structural and functional characteristics, tissue distribution and substrate. PDE8 hydrolyzes only cAMP such as PDE4 and PDE7^4^. Other families (PDE5, PDE6 and PDE9) hydrolyze only cGMP or both cyclic nucleotides (PDE1, PDE2, PDE3, PDE10 and PDE11)^4^.

The contribution of PDEs to ovarian physiology is quite striking in terms of their role in regulating the degradation of cyclic nucleotides. The current model of oocyte meiotic resumption proposes that the PDE3A expressed in the oocyte regulates cAMP concentration, which inhibits oocyte meiotic resumption^5^. This cAMP not only comes from the synthesizing ability of the oocyte but also through the permeable gap junctions between oocyte/cumulus/granulosa cells. To maintain high levels of oocyte cAMP, PDE3A activity must remain low^6^. These conditions are possible with cGMP synthesized by granulosa cells and cumulus cells^5^, since PDE3A is a cGMP-inhibited PDE^4^. In other words, the enzymatic activity of PDE3A is inhibited by high concentrations of cGMP which is synthesized through C-type natriuretic peptide stimulating NPR2 (Natriuretic peptide receptor 2) guanylyl cyclase in granulosa cells and cumulus cells^7^. Permeable gap junctions between granulosa cells, cumulus cells and the oocyte allow cyclic nucleotides to flow within this electrophysiological syncytium^5^. The response of granulosa cells to FSH and LH involves cAMP and one of the PDE roles in ovarian physiology comes from the phenotype of PDE4D knockout mice. The lack of PDE4D in granulosa cells of these female mice result in sub-fertility with low ovulation rates and altered differentiation of ovarian follicular cells^8,9^. These findings emphasize the importance of PDEs in ovarian physiology.

Mitochondria are organelles with multiple functions, including steroidogenesis. Cyclic AMP is now a known regulator of mitochondrial function^10^. The cAMP molecular machinery including adenylyl cyclases^11^, phosphodiesterases^12,13^ and protein kinases^14^ are present in mitochondria and function in supporting signal compartmentalization. Several examples argue for PDEs sub-cellular localization. As such, PDE has been found in detergent-resistant membranes such as lipid rafts, and these membrane microdomains appear to play an essential role in proper signalling^15–18^. Anchoring proteins have been found in association with PDE in sub-cellular compartments such as the Golgi apparatus, the sarcoplasma, and mitochondria. Myomegalin has been found to localize PDE4D3 in the Golgi region in cultured COS-7 cells^19^. The localization of PDE4B in sarcolemma appears to mediate the regulation of beta-adrenergic feedback in cardiac myocytes^20^. PDE3A is localized in the sarcoplasmic region in the human myocardium, where it forms a signalosome between sarcoplasmic/endoplasmic reticulum Ca^2+^-transporting ATPase 2 (SERCA2) and the anchoring protein, AKAP18^21^. The PDE2A identified from the brain and liver mitochondria of rats appear to be involved in the regulation of respiratory chain activity^12^. This is of particular interest since the mitochondrial PDE2A in cardiac myocytes has been known to regulate local cAMP levels, mitochondrial morphology and apoptosis^22^. It has also been recently reported that PDE8A and PDE8B are both involved in steroidogenesis in Leydig cells, but in different sub-cellular locations: PDE8A in mitochondria and PDE8B in the cytosol^23^. PDE8A and PDE8B both have a very high affinity for cAMP. The PDE8 family is one type of PDE that was recently discovered, known to be insensitive to the non-specific PDE inhibitor 3-isobutyl-1-methylxanthine (IBMX)^24^, and, specifically inhibited by PF-04957325^23^. Although it remains one of the least studied PDEs, we have claimed the presence of PDE8A in bovine follicular cells^25^. The aim of the present study was to assess the mitochondrial sub-cellular localization of PDE8A in ovarian follicular cells using swine as an animal model.

## Results

### PDE8A in follicular ovarian cells

**Figure 1:**
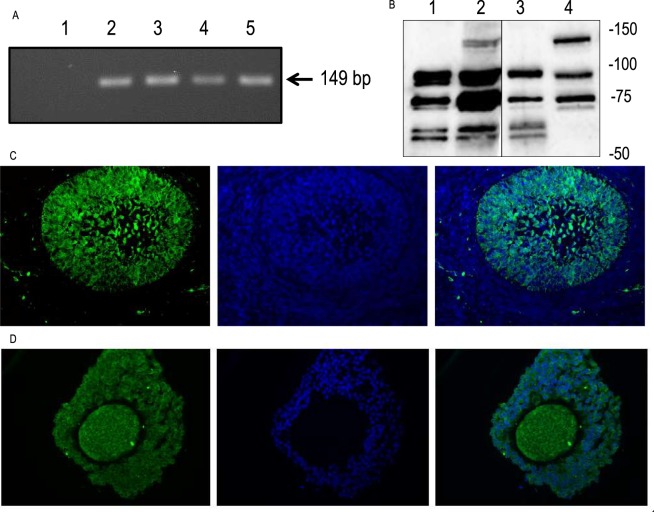
PDE8A expression in ovarian follicular cells. (**A**) PDE8A transcript detection by RT-PCR in ovarian follicular cells using PDE8A-F and PDE8A-R primers. Lane 1: PCR conducted without template; Lane 2: granulosa cells; Lane 3: COC; Lane 4: cumulus cells; Lane 5: oocytes. Amplifications were conducted on three biological replicates (n = 3). (**B**) Western blot of PDE8A in ovarian follicular cells using anti-PDE8A (Proteintech). Lane 1: granulosa cells; Lane 2: COC; Lane 3: cumulus cells; Lane 4: oocytes. Protein molecular mass markers are indicated on the right (kDa) (one representative replicate of 3). Immunohistochemical localization of PDE8A in (**C**) early antral follicle and (**D**) COC (n = 3). Immunolabelling with anti-PDE8A (Proteintech) was observed with a green signal. The DNA signal was obtained with DAPI in blue. PDE8A signal was calibrated with the non-specific IgG signal. Oocyte diameter is 100 µm.

Reverse-transcription PCR was performed on RNA extracted from granulosa cells, cumulus-oocyte complexes (COCs), cumulus cells, and oocytes using PDE8A-F and PDE8A-R primers (Table 1). This yielded an amplicon length of 149 bp based on agarose gel electrophoresis, as expected (Fig. 1A, Table 1). The sequencing revealed 99% homology with the nucleotide sequence of the porcine PDE8A gene (XM_021098897).

**Table 1:** Primers used to perform PCR amplifications.

Gene	Primer	Primer sequences	Expected size of PCR product (bp)
PDE8A	PDE8A-F	AAAGCACTTCGAGCATGTCAAC	149
	PDE8A-R	GCATTCGTTTGATCAGAGTCCG

The use of western blots revealed immuno-reactive bands in granulosa cells, COCs, cumulus cells and oocytes (in lanes 1, 2, 3, and 4, respectively, in Fig. 1B) at the molecular weights of 92, 74, 70, 58, and 56 kDa corresponding to PDE8A^15,26^. The lower two immuno-reactive bands were seen in granulosa cells, COCs and cumulus cells. Oocytes showed a distinctive band of 125 kDa. Specificity of the antibody was assessed using mouse tissue^15^ and human ovarian tissue (see Supplementary Fig. S1). In addition, immuno-reactive signals were clearly localized in granulosa cells, cumulus cells and in the oocyte (Fig. 1C,D). These results support the transcript and protein expression of PDE8A in granulosa cells, cumulus cells and the oocyte.

### The study of mitochondrial fractions

**Figure 2:**
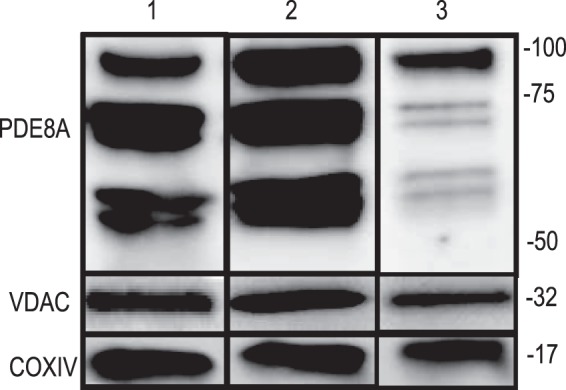
Western blot of granulosa cell protein extracts (lane 1), mitochondrial fractions from granulosa cells (lane 2), or COCs (lane 3), showing the relative abundance of PDE8A (Proteintech), voltage-dependent anion channel (VDAC, Cell signaling technology Inc.) and cytochrome c oxidase subunit IV (COXIV, Cell signaling technology Inc.). Protein molecular mass markers are indicated on the right (kDa).

The next experiments were performed to assess whether PDE8A could be detected in mitochondrial-isolated fractions. Western blots were performed on mitochondrial fractions isolated from both granulosa cells and COCs by differential centrifugation. This revealed immuno-reactive bands at molecular weights corresponding to voltage-dependent anion channels (VDAC) and cytochrome c oxidase IV (COXIV) (Fig. 2), supporting the isolation and enrichment of the mitochondria. Immuno-reactive bands corresponding to PDE8A (92, 74, 70, 58, and 56 kDa) were obtained in mitochondrial fractions from both granulosa cells and COCs, indicating the expression of PDE8A in isolated mitochondrial fractions.

The purpose of the following experiment was to assess PDE8 activity in mitochondrial fractions. IBMX-insensitive cAMP-PDE activity was measured in order to first obtain an estimate of the activity. The results showed that 53.3% of total PDE activity was measured as IBMX-insensitive cAMP-PDE activity in granulosa cell homogenates (Fig. S5). A very close percentage of 49,7% was measured in the isolated mitochondrial fractions (Fig. S5). Using the PDE8-specific inhibitor, PF-04957325, we obtained an IC_50_ of 2.5 ± 0,3 nM in granulosa cell extracts (Fig. S4), consistent with the use of an IC_90_ of 30 nM^23^. In granulosa cell extracts, 2.55 ± 0.08 picomoles of cAMP hydrolyzed per minute per million cells was measured as the total cAMP-PDE activity (Table 2). The cAMP-PDE activity that was sensitive to PF-04957325 was 1.72 ± 0.06 picomoles of cAMP hydrolyzed per minute per million cells. This suggests that PDE8 comprises more than two thirds of the cAMP-PDE activity in granulosa cells, as can be seen based on its sensitivity to PF-04957325. In isolated mitochondrial fractions from granulosa cells, the cAMP-PDE activity is one thousandth the one in granulosa cells extracts, and 77% of the activity was found to be PF-04957325-sensitive (Table 2). Thus, these data support measuring PDE8 in mitochondrial fractions as both IBMX-insensitive and PF-04957325-sensitive activities.

**Table 2:** Total and PF-04957325-sensitive PDE activities measured in granulosa cell homogenates and in mitochondria isolated fractions.

	Total PDE activity	PF-sensitive PDE activity (Percentage, %)
Granulosa cells^*^	2.55 ± 0.08	1.72 ± 0.06 (67,4%)
Isolated mitochondria^**^	1.10 ± 0.05	0.85 ± 0.05 (77,2%)

Measurements were conducted on two biological replicates in triplicate (n = 2). [PF-04957325] = 30 nM. ^*^Picomoles of cAMP hydrolyzed/min/million cells, **femtomoles of cAMP hydrolyzed/min/fraction.

**Figure 3:**
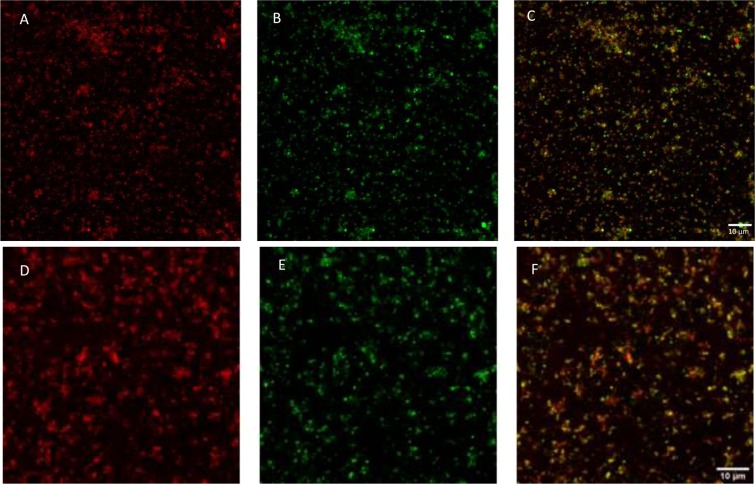
Immunolocalization in mitochondrial isolated fractions from granulosa cells by immunofluorescence using anti-P450scc (green, **B**, United States Biological), anti-PDE8A (green, **E**, Proteintech) and MitoTracker (orange, **A**,**D**). Merge signals are shown in (**C**,**F**). The signal was calibrated with non-specific IgG. Magnification is shown as a bar of 10 µm.

Labelling mitochondrial fractions with MitoTracker Orange CMTMRos clearly supports mitochondrial enrichment (Fig. 3A). Using immunofluorescence with P450scc antibody (an exclusive mitochondrial enzyme in granulosa cells) together with MitoTracker revealed an overlapping signal (Fig. 3C). Mitochondrial fractions were submitted to immunofluorescence with antibody specific for PDE8A, an overlap was observed between the green immunofluorescence labelling for PDE8A and the orange staining from MitoTracker (Fig. 3F). This overlap further supports PDE8A localization at the mitochondrial sub-cellular compartment.

**Figure 4:**
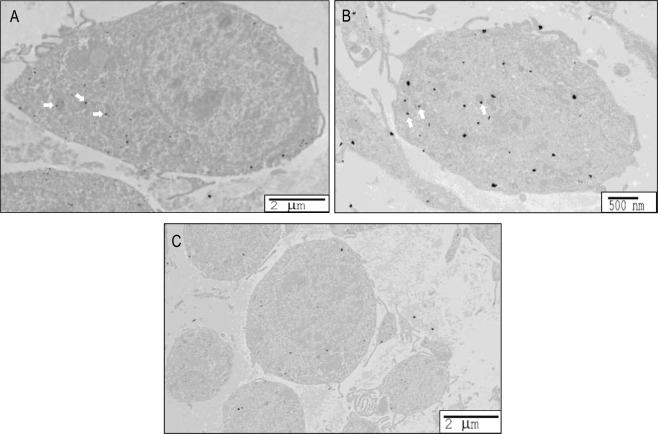
PDE8A sub-cellular localization in cumulus cells (**A**,**B**) was revealed by immunoelectron microscopy. After isolation COCs were fixed in acrolein and thick slices were incubated with anti-PDE8A. Immunolabelling of PDE8A with mitochondria were marked by arrows. Negative control with IgG is shown in (**C**).

The next experiment conducted used immunoelectron microscopy to assess mitochondrial PDE8A sub-cellular localization. PDE8A immunostaining was observed at the plasma membrane, in the cytosol, and was associated with the mitochondria of cumulus cells in COCs (Fig. 4A,B). In order to confirm immunostaining specificity, two sets of negative controls were used. In the first, the primary antibody was replaced with an equal concentration of immune IgG. In the second, the silver enhancement was performed alone (Fig. 4C).

Effect of PDE8 inhibition on progesterone secretion and active mitochondria during *in-vitro* maturation (IVM)

**Figure 5:**
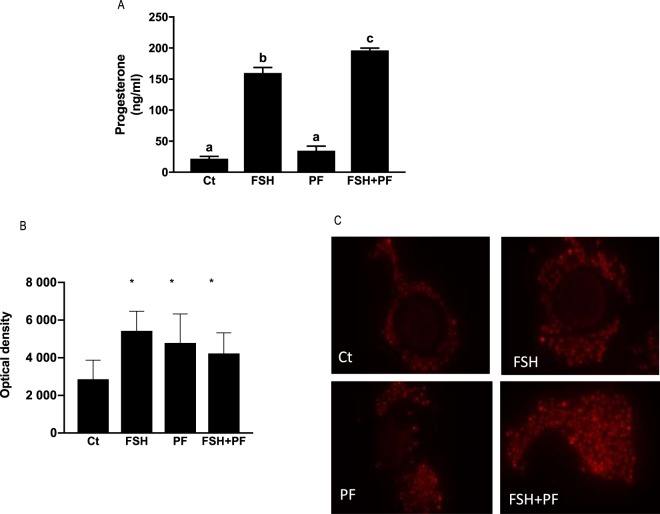
Effect of PDE8A inhibition on (**A**) progesterone synthesis and (**B**) active mitochondria in cumulus cells during *in-vitro* maturation of COC, for 48 h in IVM medium, without stimulation (Ct), with recombinant human FSH (FSH), with PF-04957325 (specific PDE8 inhibitor, PF) or with FSH and PF-04957325 (FSH + PF). (**A**) Progesterone was assayed in triplicate in three biological replicates (n = 3). Different letters indicate statistically significant differences (P < 0.05). (**B**) Active mitochondria were measured in cumulus cells using MitoTracker. Asterisk indicates statistical significance (P < 0.05) with the control. (**C**) Representative images of active mitochondria measured in cumulus cells using MitoTracker orange CMTMRos.

In order to evaluate the potential role of PDE8 in the steroidogenesis of cumulus cells, COCs were treated with a PDE8-specific inhibitor, PF-04957325 (300 nM)^23^, during IVM. Then, the amount of progesterone in the medium was quantified by enzyme immunoassay. COCs responded to gonadotropins by synthesizing progesterone during IVM, as it has already been demonstrated^27^. When recombinant human FSH was present, PF-04957325 significantly increased progesterone secretion compared to when there was no inhibitor. The absence of the recombinant human FSH showed no significant change, with or without PF-04957325 (Fig. 5A). These results indicate that inhibiting PDE8 significantly regulated FSH-stimulated progesterone secretion during IVM.

Active mitochondria were analysed in histological sections of the treated COCs using MitoTracker orange CMTMRos (Fig. 5C). The optical density analysis revealed significant increased by recombinant human FSH, by the specific PDE8 inhibitor, PF-04957325, and both (Fig. 5B). The increase in active mitochondria by PF-04957325 supports a functional regulation of PDE8 at the mitochondrial sub-cellular compartment.

## Discussion

This study indicates that PDE8A is both expressed and functional in the granulosa and cumulus cells of the ovarian follicle. Sub-cellular localization of PDE8A is also suggested by the following observations. Mitochondrial isolated fractions showed immuno-reactive bands through western blot techniques, showed both PDE8 IBMX-insensitive and PDE8 PF-04957325-sensitive cAMP-PDE activity, and were immuno-reactive to PDE8A specific antibody. The subcellular localization of PDE8A was also supported by immunoelecton microscopy, which showed immunostaining for PDE8A associated with mitochondria. During IVM, FSH-stimulated progesterone secretion from cumulus cells was significantly regulated by the specific inhibition of PDE8. Active mitochondria were increased by the specific PDE8 inhibition.

FSH-stimulated progesterone secretion has been previously observed in granulosa cells and COC^28,29^. Specific inhibition of PDE8 by PF-04957325 resulted in a significant increase in progesterone secretion when stimulated by FSH. An increase in progesterone secretion by IBMX has been reported when granulosa cells were treated with FSH^29^. Interestingly, FSH-induced progesterone secretion in human cumulus granulosa cells was decreased by a common herbicide, atrazine^30^. This environmental contaminant alters steroidogenesis by decreasing cAMP through an increase in cAMP-PDE activity^30^, supporting the involvement of phosphodiesterase in progesterone secretion.

Recent studies have reported that granulosa cells from human expressed both PDE8A and PDE8B^31^. In both COCs and granulosa cells from cattle, IBMX-insensitive cAMP-PDE activity was observed^25^. In cumulus and granulosa cells, both PDE8A and PDE8B were present^25^. In swine, a recent study showed IBMX-insensitive cAMP-PDE activity in the detergent-resistant membrane (DRM)^15^ of granulosa cells, suggesting the presence of an active PDE8 in membrane microdomains. Although this PDE8 activity was not exclusive to DRM, only PDE8A was further studied using western blot^15^.

Different roles and functions have been proposed for PDE8 depending on which tissues it is present in. It has been hypothesized that the PDE8A gene may play a role in polycystic ovary syndrome in humans (PCOS). The hypothesis is based on the idea that reduced PDE8A expression or activity in theca cells of the ovarian follicle could contribute to excessive androgen production^32^. Despite the identification of new PDE8A variants and their localization in the plasma membrane of theca cells, little evidence is available about how genetic variations in PDE8A affect the risk of developing PCOS. The involvement of PDE8 in testosterone production by Leydig cells^33^ is noteworthy. Based on responses to the PDE8-specific inhibitor PF-04957325 and to gene null mutation, PDE8A and PDE8B (both strongly expressed) have been found to suppress resting steroidogenesis (including testosterone synthesis)^23^. PDE8A has been observed to co-localize with mitochondrial P450scc in Leydig cells^19^, as seen in isolated mitochondrial fractions from granulosa cells in this study (Fig. 3C).

Mitochondrial function can be modulated by calcium and cAMP. The null mutation of PDE8A gene in mice potentiates cAMP/PKA-elicited increases in L-type Ca^2+^ channel current and sarcoplasmic Ca^2+^ release during beta-adrenergic stimulation^34^. The transport of Ca^2+^ modulates various aspects of mitochondrial function^35^ such as an increase in ATP production and activation of mitochondrial metabolism^36^. As explained in a recent review, cAMP signalling is recognized as a modulator for mitochondrial dynamics^10^. It appears that mitochondrial PDE2A regulates respiratory metabolism^12^ in rodent liver and brain tissue. In the present study, less than 20% of cAMP-PDE activity was EHNA-sensitive (PDE2-sensitive) in isolated mitochondria from granulosa cells (data not shown). When using gene null mutation in *Drosophila*, the cAMP-PDE Prune localized in the mitochondrial matrix was found to play a role in mitochondrial biogenesis^13^.

One way of anchoring PDE to membranes involves N-myristoylation. This process appears to take place for human PDE8A^26^. There is a 92% sequence homology between swine PDE8A and its human counterpart, which indicates that they contain similar N-myristoylation sequences. This was validated using Myristoylator (https://web.expasy.org/myristoylator/). PDE8A also contains a Per-ARNT-Sim (PAS) domain^37^. This is a structural motif and a protein sensor domain that is involved in various biological processes, such as responding to partial pressure changes in oxygen and redox signalling. In addition, it has been demonstrated that PDE8 may form a complex with the regulatory subunit of PKA channeling cAMP degradation to facilitate cAMP desensitisation^38^. The mitochondrial subcellular localization of PDE8A suggests its contribution to finely tuned mitochondrial function such as steroidogenesis.

In conclusion, the role of cAMP regulation in ovarian follicular cells must be better understood in order to improve artificial reproductive technologies for livestock producers and would-be parents. Improving the quality of the oocyte obtained from ovarian stimulation and/or IVM remains one of the greatest challenges of artificial reproduction. The involvement of molecular devices such as PDE8A and its mitochondrial sub-cellular localization may have implications in steroidogenesis, oocyte physiology and obtaining oocyte developmental competence. Further investigation is required.

## Materials and Methods

### Chemicals

Unless otherwise stated, all chemicals were purchased from Sigma Chemical Company (St. Louis, MO, USA).

### Ovary collection and tissue recovery

As described previously, pre-pubertal pig ovaries were collected^39^. Briefly, the ovaries were recovered from a local slaughterhouse, placed in saline solution (0.9% NaCl) containing antibiotics and antimycotics (100,000 IU/L penicillin G, 100 mg/L streptomycin, 250 μg/L amphotericin B) and maintained at 37 °C. On arrival at the laboratory, they were rinsed in saline solution containing antibiotics and antimycotics at 37 °C. Antral follicles (3 to 6 mm) were punctured using an 18-gauge needle attached to a 10 mL syringe in order to collect a mixture of follicular cells (cumulus-oocyte complexes and granulosa cells) in follicular fluid. Granulosa cells and cumulus-oocyte complexes were selected according to the criteria described previously^40^. They were then washed with HEPES buffered Tyrode medium containing 0.01% (w/v) polyvinyl alcohol (PVA-HEPES)^41^. The cells were used immediately or flash-frozen for future use depending on the experiment.

### RT-PCR

Total RNA was isolated from several sheets of granulosa cells, 50 COC, cumulus cells obtained from 50 COCs and 50 oocytes denuded of cumulus cells (DO) using the PicoPure™ RNA Isolation Kit. RNA samples were suitably diluted in elution buffer after determining concentration and purity using a Nanodrop bioanalyser (ThermoFisher Scientific, Waltham, MA). Total RNA was reverse transcribed using the qScript Flex cDNA Kit from Quanta Biosciences (Beverly, MA). The primer pairs designed for the porcine PDE8A sequence (shown with the PCR product size in Table 1) were purchased from Integrated DNA Technologies (Coralville, IA). The cDNA was amplified using an AccuStart II PCR SuperMix (2X) kit from Quanta Biosciences. Negative controls were treated in parallel and under the same conditions in order to detect residual contamination with genomic DNA. PCR products were visualized via electrophoresis using 2% agarose gel stained with EZ vision DNA dye (VWR, Ville Mont-Royal, Québec). All experiments were done in triplicate. The PCR products were then purified for sequencing at the Plateforme de Séquençage et de Génotypage des Génomes (CHUL) in order to confirm their homology with porcine PDE8A (XM_021098897).

### Western blotting

Proteins were extracted from granulosa cells using hypotonic buffer (TRIS-HCl 20 mM pH 7.4, EDTA 1 mM, EGTA 0.2 mM, sodium fluoride 50 mM, benzamidine 50 mM, sodium pyrophosphate 10 mM, aprotinin 4 µg/mL, pepstatin 0.7 µg/mL, soybean trypsin inhibitor 10 µg/mL, leupeptin 0.5 µg/mL and phosphatase inhibitor phenylmethylsulfonyl fluoride (PMSF) 2 mM). The total protein and mitochondrial protein isolated from follicular cells (20 µg of granulosa cell protein extract, 50 COCs, cumulus cells obtained from 50 COCs, 50 oocytes denuded of cumulus cells (DO) and mitochondrial fractions isolated from granulosa cells and COCs (see below) and protein standards 161-0374 (BioRad, Hercules, CA)) were loaded onto 7.5% SDS-polyacrylamide gel for electrophoresis. The proteins were then transferred onto polyvinylidene difluoride (PVDF) membranes, which were blocked for 60 min with TBS containing 0.1% (v/v) Tween-20 and 5% (w/v) non-fat dry milk. Blots were then treated at 4 °C overnight with primary antibodies. These primary antibodies were namely anti-PDE8A (Proteintech, Rosemont, IL, cat no. 13956-1-AP), anti-VDAC (Cell signaling technology Inc., Danvers, MA, USA, #4866S) and anti-COXIV (Cell signaling technology Inc., Danvers, MA, USA, #4850S). All primary antibodies were diluted by a factor of 1:1 000. The membranes were then blotted for 1 h with horseradish-peroxidase-conjugated secondary antibody Goat Anti-Rabbit IgG (Invitrogen, 1:20 000) and proteins were detected by chemiluminescence, using the Clarity Western ECL Substrate detection system from Bio-Rad (Mississauga, Ontario, Canada, cat no. 170–5061). Images were obtained using a Fusion FX7 reader from Vilber-Lourmat Lab Equipment (Montreal Biotech Inc., Dorval, CA) with Fusion software. Bio-1D software (Montreal Biotech Inc) was used for image analysis.

### Immunohistochemistry

Immunohistochemistry was performed on sections of ovarian pieces and COCs. Pieces of tissues were rapidly fixed in Bouin’s fixative solution for 1 h at room temperature while COCs were fixed in 4% (w/v) PFA for 10 min at room temperature. Both of them were dehydrated in four increasing concentrations of alcohol baths and impregnated with hot paraffin (three baths at 44–60 °C) to solidify the tissue. Samples enrobed with paraffin were then cut into 5 μm sections using a microtome (Microm HM330 Heidelberg, Germany) and sections were mounted on slides prior to use. The slides were deparaffinized, rehydrated in ethanol baths and washed in PBS solution. Endogen peroxidase activity was inhibited with 3% hydrogen peroxide in methanol for 10 min and antigen retrieval was achieved by boiling the slides for 20 min in Tris 10 mM/EDTA 1 mM pH 9.0. Non-specific sites were blocked with 1% BSA in PBS for 20 min, slides were next incubated overnight (4 °C) with a primary antibody anti-PDE8A (1:200) and the immune complex was revealed after incubation with Alexa Fluor 488 Goat Anti-Rabbit IgG secondary antibody (Invitrogen, 1:200) for 1 h. Nonspecific labelling was assessed using an equivalent concentration of non-PDE8A-immune IgG (Jackson ImmunoResearch Laboratories Inc., West Grove, PA, USA). Before putting a coverslip, cell nuclei were stained with ProlongGold anti-fade containing 40,6-diamidino-2-phenylindole (DAPI; Invitrogen). Images were acquired on a Nikon Eclipse TE2000-E inverted confocal microscope (Nikon, Mississauga, ON, Canada) at 40 × magnification.

### Assessment of active mitochondria

Active mitochondria in cumulus cells were assessed according to the treatments for progesterone quantification. After IVM, COCs were labelled using 500 nM of MitoTracker Orange (CMTMRos, ThermoFisher Scientific, Waltham, MA) for 30 min at 37.5 °C. The samples were washed twice in PBS solution and fixed with 4% (w/v) PFA for 10 min in the dark at 37 °C. After fixation, COC were prepared for paraffin section as described above. From 5 µm sections stored in dark at 4 °C, epifluorescent images were obtained with a Zeiss Axio Observer.Z1 epifluorescence microscope using widefield illumination (Collibri.2 at 530 nm and rhodamine filter) with a 40x/0.95NA objective (Carl Zeiss Canada Ltd., Toronto) and a Zeiss AxioCam MRm camera. The focus was made on the brighten fluorescent plane and the acquisition time (200 ms) was kept constant for all experiments.

### Isolation of mitochondria

Mitochondria were isolated from 1 000 COCs and 4 millions of granulosa cells by differential centrifugation after cell lysis^42^. Individual granulosa cells were obtained by gently disrupting sheets of tissue using a micropipette and counted using a NEUBAUER type hemocytometer. The cells were washed twice by centrifuging (1500 × *g* for 2 min) in 1 mM Tris-HCl buffer (pH 7.0) containing 0.13 M of NaCl, 5 mM of KCl and 7.5 mM of MgCl_2_. The cells were then mechanically triturated in 3.5 mM Tris-HCl buffer (pH 7.8) containing 2 mM of NaCl and 0.5 mM of MgCl_2_ (half of the cell pellet volume). The cell homogenate was immediately thinned with the 3.5 mM Tris-HCl buffer, diluted 10-fold. After differential centrifugation (13,000 × *g* for 1 min), the mitochondria were washed twice by gentle pipetting in 35 mM Tris-HCl buffer (pH 7.8) containing 20 mM of NaCl and 5 mM of MgCl_2_ and centrifuged (13,000 × g for 1 min). The pellet was either immediately used or stored at −80 °C according to the experiments. The entire procedure was performed at 4 °C.

### Immunofluorescence

To immunostain mitochondrial PDE8A, freshly pelleted mitochondria were suspended in 1:1 ratio of Tris-HCl buffer pH 7.8 and were labelled using 500 nM of MitoTracker Orange (CMTMRos, ThermoFisher Scientific, Waltham, MA) at 38 °C. The samples were washed twice in PBS solution, centrifuged at 14,000 × *g* for 5 min to eliminate residual markers, fixed with 4% (w/v) PFA in 0.2% Triton X-100 for 10 min in the dark at room temperature, blocked with 5% BSA in PBS for 1 h, then kept at 4 °C overnight with PDE8A polyclonal antibody (Proteintech product number 13956-1-AP, 1:500 in PBS) or porcine P450scc polyclonal antibody (United States Biological, Salem, MA, product number 362774, 1:100 in PBS). The immune-reactive signal (PDE8A or P450scc) was visualized using Alexa Fluor 488 Goat Anti-Rabbit IgG secondary antibody (Invitrogen, 1:200). Non-specific labelling was assessed using an equivalent concentration of non-immune IgG (Jackson ImmunoResearch Laboratories Inc., West Grove, PA, USA). All the samples were mounted on glass slides using Grace Bio-Labs 200 SecureSeal imaging spacers (9 mm dia. × 0.12 mm). Fluorescent images were obtained with a Zeiss LSM 700 confocal microscope using ZEN capture at 63X magnification, 1.4 numerical aperture (NA) Plan apo VC oil immersion objective and image analysis software (Carl Zeiss Canada Ltd, Toronto, Canada). The signal was calibrated with non-specific labelling.

### PDE assay

PDE8 activity in porcine granulosa cells and mitochondria was measured with 1 μM of cAMP present at 34 °C, following the method described previously^43^. The granulosa cells were lysed in hypotonic buffer^25,44^. The assay was carried out in 200 μL (final volume) of 40 mM Tris-HCl buffer (pH 8.0) containing 10 mM of MgCl_2_, 5 mM of 2-mercaptoethanol, 0.75 mg/ml of BSA (Fraction V) and 1 μM of cold cAMP plus 15 nM of [^3^H] cAMP (1 × 10^5^ cpm/tube, 30 Ci/mmol, GE Healthcare, Baie d’Urfé, QC, Canada). Following incubation with 5′-nucleotidase, adenosine was purified by anion-exchange chromatography followed by quantification using liquid scintillation counter (Perkin Elmer Winspectral 1414, Woodbridge, Ont). The PDE activity was measured both with and without PDE8 inhibitor PF-04957325 (30 nM), and with IBMX (500 mM). The concentration of PF-04957325 that was used in the assay was calculated from the enzyme activity IC_50_ based on a dose response curve (Fig. S4), and is in accordance with previously used values^23^. Separate experiments were performed in triplicate for each enzyme assay.

### Immunoelectron microscopy

A well-defined protocol^45^ was adapted for ovarian cells. Fifty fresh COCs were fixed directly in 0.5 mL of 3.5% acrolein for 30 min at room temperature, then washed twice in PBS to remove the excess fixative. After a quick centrifugation, cell pellets (COC) were gently mixed with 125 µL of 4% agarose and placed at 4 °C until solid, and then cut into 50 μm sections using a vibratome (Leica VT1000S, Leica Biosystems, Concord, Ont). These were washed three times in PBS for 10 min each, held in 0.1% sodium borohydride (NaBH_4_) in PBS for 30 min, and washed again in PBS (three times for 10 min). They were then blocked for 2 h in PBS containing 10% fetal bovine serum, 3% BSA and 0.01% Triton X-100 and incubated with anti-PDE8A (Proteintech product number 13956-1-AP, 1:500 in blocking solution) overnight at 4 °C. Next, they were washed five times in TBS (5 min each) before incubating the sections with the secondary antibody (1:100 in TBS; gold-coupled (1.4 nm particles) goat anti-rabbit IgG, Nanoprobes, Yaphank, NY, USA) overnight at 4 °C. The sections were then washed three times for 5 min each with TBS, followed by two washes (5 min each) with 3% sodium acetate. Using a gold enhancement kit (HQ Silver, Nanoprobes, Yaphank, NY), the staining was revealed at room temperature for 1 min, rinsed quickly with sodium acetate solution, then rinsed three times for 5 minutes each with PBS. Post fixation of the COCs sections was achieved with osmium tetroxide. The sections were then dehydrated with sequential alcohol baths and propylene oxide. As described previously^46^, the sections were embedded in Durcupan resin between two ACLAR sheets and placed in the oven at 55 °C for 3 days. Ultrathin sections of ~70 nm were generated from the region of interest of the 50 μm sections using a Leica UC7 ultramicrotome. Images were acquired using a FEI Tecnai Spirit G2 transmission electron microscope (Thermo Fisher Scientific Company, Hillsboro, OR) at 80 kV.

### Progesterone quantification

As previously described, a commercial immunoassay (ALPCO Diagnostics, Salem, NH, product number 11-PROHU-E01) was used^27^ to measure the progesterone secreted by porcine COCs cultured in North Carolina State University-23 (NCSU-23) medium^47^ without bovine serum albumin containing 25 µM of 2-mercaptoethanol, 0.1 mg/ml of cysteine, 10% (v/v) filtered porcine follicular fluid, and 0.01 µg/ml of recombinant human FSH (GONAL-f, Serono, Mississauga, ON, Canada)^47^. This was done in the presence or absence of the PDE8 inhibitor (PF-04957325, donated by Pfizer under a Material Transfer Agreement). The concentration of PF-04957325 (300 nM) corresponded to the cell based IC_90_^23^. For each experimental condition, 10 COCs were incubated for 48 h in an equal volume (125 µL) of medium in 96-well assay plates. Maturation medium and COCs were recovered from three independent experiments and kept at −80 °C until use. The progesterone in the maturation medium was assayed in triplicate according to the manufacturer’s instructions. The sensitivity of the assay was 0.1 ng/mL.

### Statistical analysis

All means are presented with their corresponding SEM. Statistical analysis was performed using GraphPad Prism 8.0.1 for MacOS (GraphPad Software Inc., San Diego, CA). Statistical significance was assessed by one-way ANOVA, followed by either Dunnett’s or Bonferronni’s multiple comparison post hoc tests in order to identify individual differences between means. Probabilities of P < 0.05 were considered statistically significant.

### Third party rights

The PDE8-specific inhibitor, PF-04957325, was donated by Pfizer under a Material Transfer Agreement.

## Supplementary information

Supplemental information

## Data Availability

All data generated during this study are included in this published article.
