# Supplementary material for: Mitochondrial sub-cellular localization of cAMP-specific phosphodiesterase 8A in ovarian follicular cells
Source: Sci Rep. 2019 Aug 28;9:12493. doi: 10.1038/s41598-019-48886-8 (PMC6713761; doi:10.1038/s41598-019-48886-8)

Mitochondrial sub-cellular localization of cAMP-specific phosphodiesterase 8A in ovarian follicular cells.

Amel Lounas[1], Nathalie Vernoux[2], Marc Germain[3], Marie-EveTremblay[2], François J Richard[1]*

*Correspondance : francois.richard@fsaa.ulaval.ca

Supplementary Figures

Figure S1: Western blot of human ovarian tissue (lane 1) and porcine granulosa cells (lane 2) showing the presence of PDE8A.

Figure S2: The original western blot of figure 1B for PDE8A using anti-PDE8A (Proteintech). Lane 1: granulosa cells; Lane 2: COC; Lane 3: cumulus cells; Lane 4: oocytes. Protein molecular mass markers are indicated on the right (kDa).

Figure S3: The original WB of figure 2: for PDE8A. Lane 1: granulosa cells extract; Lane 2: Mitochondrial enriched fraction from granulosa cells; Lane 3: Mitochondrial enriched fraction from COCs. Western blots using anti-PDE8A (Proteintech), anti-voltage-dependent anion channel (VDAC, Cell signaling technology Inc.) and anti-cytochrome c oxidase subunit IV (COXIV, Cell signaling technology Inc.) are shown. VDAC and COXIV were both hybridized in the same time. Protein molecular mass markers are indicated on the right (kDa).

Figure S4: cAMP-PDE activity measured in presence of increasing concentration of PF04957325 inhibitor in granulosa cell homogenates (fmoles of cAMP hydrolyzed/min/fraction). Measurements were conducted in two biological replicates in triplicate (n = 2). The calculated IC_{50} was 2.8 and 2.2 nM for replicate #1 and #2, respectively. The IC_{90} was estimated to be 25 nM based on the IC_{50} mean.

Figure S5: Total and IBMX-sensitive cAMP-PDE activity measured in granulosa cells homogenates (fmoles of cAMP hydrolyzed/min/microgramme of protein) and in mitochondria isolated fractions (fmoles of cAMP hydrolyzed/min/fraction). Measurements are from triplicate and plotted as dots for each replicate and as mean +/- SEM for the three biological replicates (n = 3). The percentage of IBMX-insensitive activity was calculated for each biological replicate (dots) and plotted on the graph as mean +/- SEM.

Figure S6: Effect of specific PDE8 inhibition using PF-04957325 inhibitor on A) the percentage of meiotic resumption measured as germinal vesicle breakdown (GVBD), and B) cumulus cells expansion after 48h of IVM in presence and absence of FSH stimulation.

Supplementary Figure S1

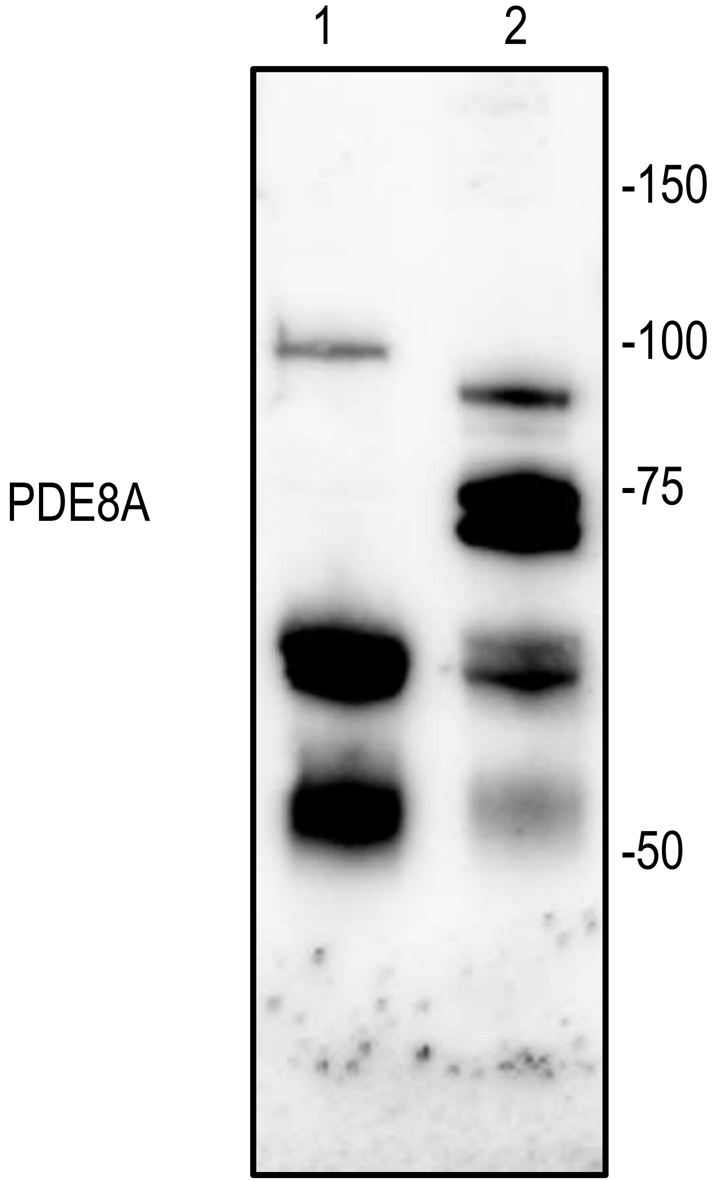

Supplementary Figure S2

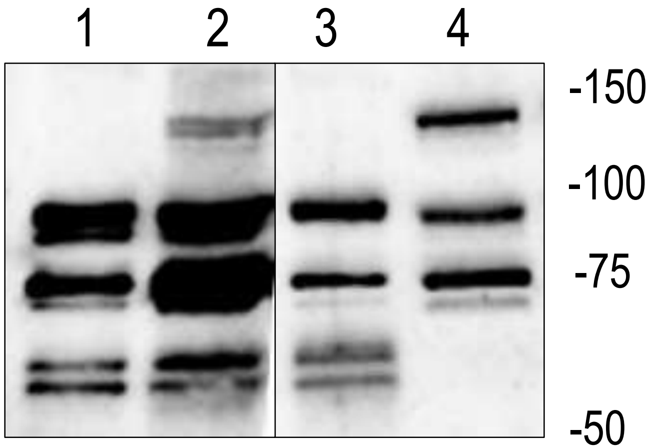

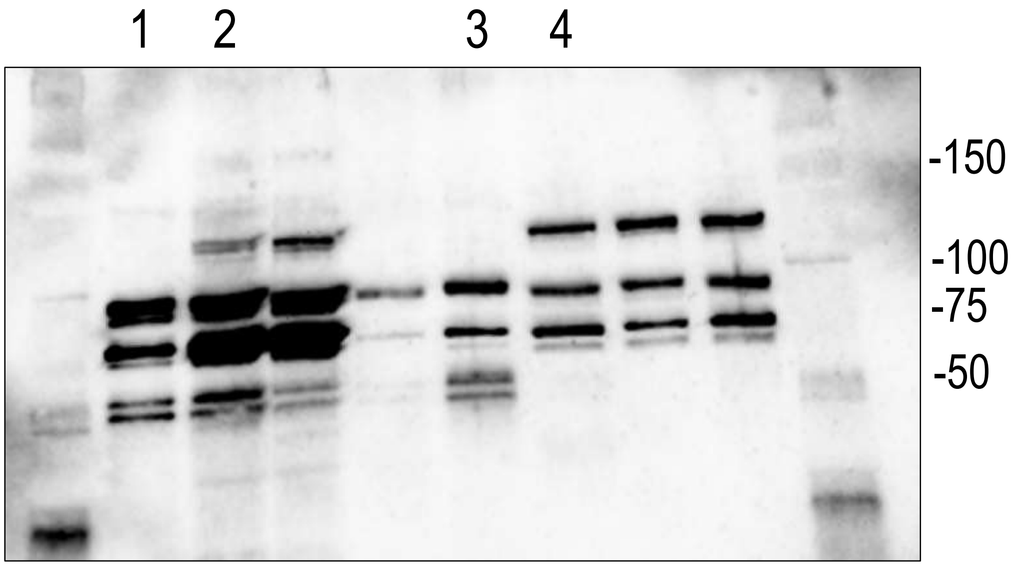

Supplementary Figure S3

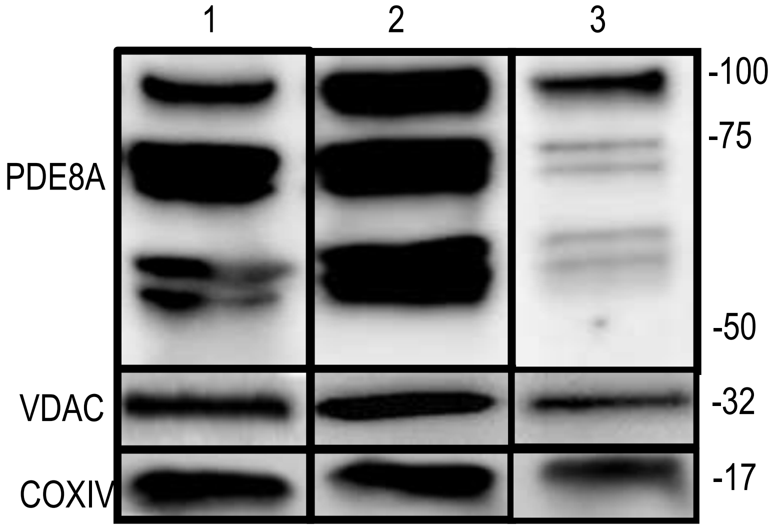

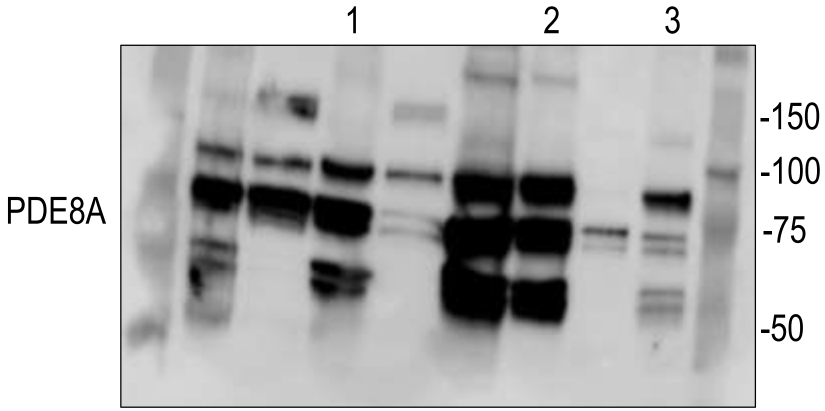

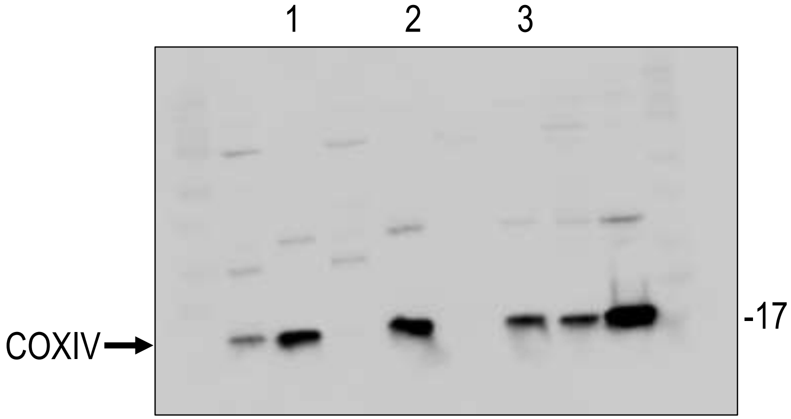

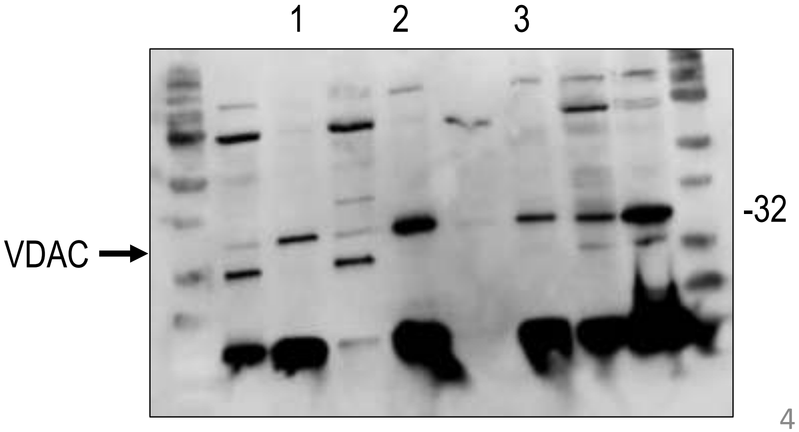

Supplementary Figure S4

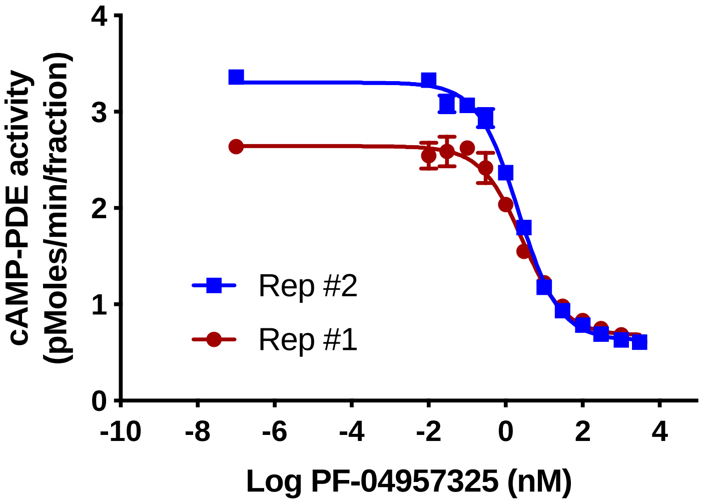

Supplementary Figure S5

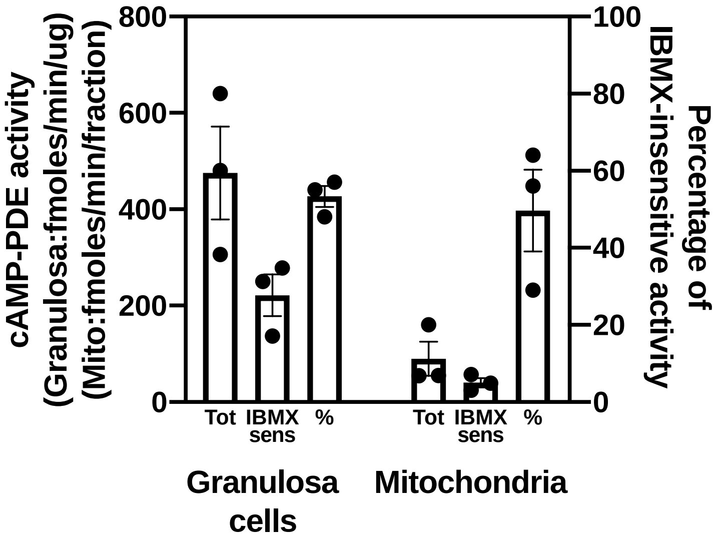

Supplementary Figure S6

A

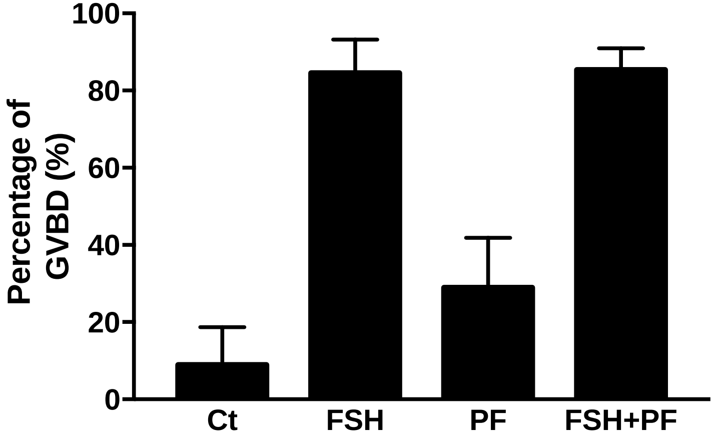

B

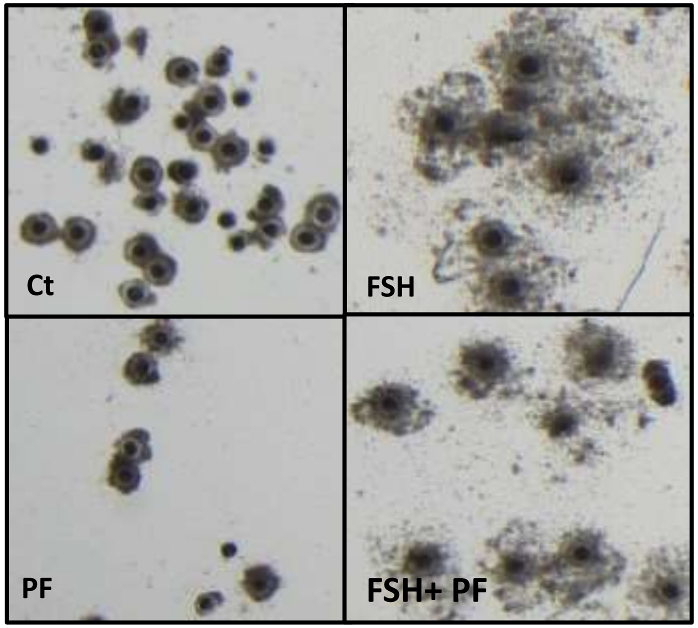

Supplement: Supplementary file 1 — Supplemental information [file 41598_2019_48886_MOESM1_ESM.pdf]
